# Supplementary material for: Characteristic Volatile Fingerprints of Four Chrysanthemum Teas Determined by HS-GC-IMS
Source: Molecules. 2021 Nov 24;26(23):7113. doi: 10.3390/molecules26237113 (PMC8658894; doi:10.3390/molecules26237113)
Supplement: Supplementary file 1 [file molecules-26-07113-s001.zip › molecules-1439937-supplementary.pdf]

Supplementary Materials

Characteristic Volatile Fingerprints of Four Chrysanthemum Teas Determined by HS-GC-IMS

Zhiling Wang, Yixin Yuan, Bo Hong, Xin Zhao - and Zhaoyu Gu -

Beijing Key Laboratory of Development and Quality Control of Ornamental Crops, Department of Ornamental Horticulture, China Agricultural University, Beijing 100193, China; bs20193170894@cau.edu.cn (Z.W.); yyxyuanyi@cau.edu.cn (Y.Y.); hongbo1203@cau.edu.cn (B.H.)

- Correspondence: zhaoxin5691@cau.edu.cn (X.Z.); 2019125@cau.edu.cn (Z.G.); Tel:86-010-62732641 (X.Z. & Z.G.)

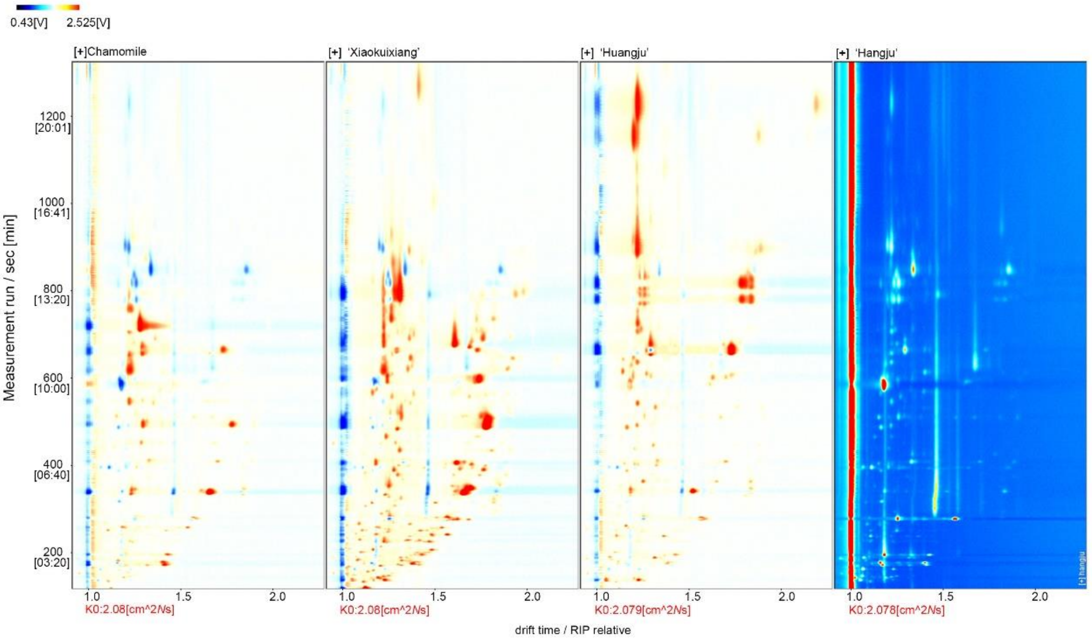

Figure S1. Gas-phase ion mobility spectra of VOCs from four chrysanthemum teas. The coloring conveys the substance's signal intensity: white indicates low intensity, and red indicates high intensity; hence, the darker the color, the higher the intensity.

Table S1. Total volatile compounds were identified from the four chrysanthemum teas.

Compound	CAS	Formula	MW	RI	Rt [sec]	Dt [a.u.]
1	-	-	-	578.2	138.421	1.04939
2	-	-	-	578.2	138.421	1.2297
3	-	-	-	588.7	143.368	1.02412
4	-	-	-	588.7	143.368	1.19178
5	-	-	-	593.9	145.841	1.22211
6	-	-	-	608	152.519	0.95082
7	-	-	-	611.6	154.25	1.27014
8	-	-	-	617.9	157.218	1.13786
9	-	-	-	641.9	168.594	1.03254
10	-	-	-	647.2	171.067	1.20695
11	-	-	-	674.3	183.928	1.08225
12	-	-	-	671.7	182.691	1.32153
13	-	-	-	686.3	189.616	1.0713
14	-	-	-	686.8	189.863	1.31479
15	-	-	-	687.3	190.111	1.34681
16	-	-	-	684.2	188.627	0.94829
17	-	-	-	703.1	202.879	1.10113

18	-	-	-	717.4	214.999	1.20563
19	-	-	-	735.3	230.018	1.09242
20	-	-	-	734.3	229.228	1.35694
21	-	-	-	734	228.964	1.4179
22	-	-	-	749.3	241.844	1.4842
23	-	-	-	751	243.295	1.18136
24	-	-	-	761.5	252.11	1.10776
25	-	-	-	759.6	250.47	1.42516
26	-	-	-	773.4	262.154	1.15721
27	-	-	-	779.5	267.279	1.08821
28	-	-	-	778.1	266.049	1.36766
29	-	-	-	789.6	277.528	1.61952
30	-	-	-	789.3	277.118	1.68277
31	-	-	-	815.1	307.251	1.12616
32	-	-	-	816.2	308.481	1.28371
33	-	-	-	822.4	315.666	1.15996
34	-	-	-	821.6	314.73	1.50383
35	-	-	-	831.2	325.962	1.31437
36	-	-	-	830.2	324.792	1.82694
37	-	-	-	833.4	328.536	1.25209
38	-	-	-	835.2	330.642	1.18072
39	-	-	-	854.9	353.574	1.25079
40	-	-	-	854.5	353.106	1.59077
41	-	-	-	863.4	363.402	1.65176
42	-	-	-	864.2	364.338	1.25209
43	-	-	-	864.4	364.572	1.83602
44	-	-	-	887.3	391.248	1.27804
45	-	-	-	887.5	391.482	1.76725
46	-	-	-	852.3	350.504	1.68933
47	-	-	-	892.2	397.015	1.06302
48	-	-	-	891.9	396.592	1.11246
49	-	-	-	889.5	393.841	1.63988
50	-	-	-	896.4	404.635	1.14872
51	-	-	-	897.2	406.116	1.18168
52	-	-	-	898	407.618	1.61492
53	-	-	-	913	435.292	1.30685
54	-	-	-	912.9	434.997	1.80988
55	-	-	-	915.1	439.119	1.20267
56	-	-	-	919.6	447.362	1.30982
57	-	-	-	922.6	452.85	1.28323
58	-	-	-	922	451.788	1.85924
59	-	-	-	929.9	466.385	1.12691
60	-	-	-	930.4	467.181	1.23676
61	-	-	-	930.1	466.65	1.7339
62	-	-	-	941	486.82	1.16493
63	-	-	-	944.2	492.682	1.21305
64	-	-	-	943.8	491.878	1.2993
65	-	-	-	947.7	499.112	1.78388
66	-	-	-	957.8	517.599	1.31812
67	-	-	-	973.9	547.339	1.22246
68	-	-	-	975.2	549.75	1.29303
69	-	-	-	985.3	568.237	1.16287
70	-	-	-	1004.1	602.934	1.62905
71	-	-	-	1002.3	599.641	1.74033
72	-	-	-	1011.3	616.196	1.14231
73	-	-	-	1013.5	620.289	1.21885
74	-	-	-	1019.5	631.309	1.19074
75	-	-	-	1014.9	622.808	1.35475
76	-	-	-	1006.5	607.38	1.29383
77	-	-	-	1010.1	613.992	1.40318
78	-	-	-	1015	623.123	1.89523
79	-	-	-	1026.5	644.218	1.14231

80	-	-	-	1027.7	646.422	1.36881
81	-	-	-	1027.8	646.737	1.92491
82	-	-	-	1032.6	655.553	1.2251
83	-	-	-	1038.4	666.258	1.26728
84	-	-	-	1045.9	680.111	1.60781
85	-	-	-	1053.3	693.753	1.90303
86	-	-	-	1068.2	721.373	1.28066
87	-	-	-	1107.4	793.817	1.31334
88	-	-	-	1106.7	792.459	1.94037
89	-	-	-	1101.6	782.951	1.78789
90	-	-	-	1100.6	781.14	1.83924
91	-	-	-	1120.2	817.362	1.78945
92	-	-	-	1119	815.098	1.83924
93	-	-	-	1120.7	818.268	1.16397
94	-	-	-	1136.8	848.151	1.33356
95	-	-	-	1136.8	848.151	1.8548
96	-	-	-	1163.2	896.806	1.36109
97	-	-	-	1302.5	1154.199	1.19927
98	-	-	-	1303.2	1155.48	1.8791
99	-	-	-	1342.3	1227.819	1.22152
100	-	-	-	1341.7	1226.539	2.19069
101	-	-	-	1364.9	1269.43	1.41374
102	-	-	-	1221.3	1004.181	1.4941
103	-	-	-	1263.1	1081.458	1.36605
(*E*)-2-Hexenal dimer	C6728263	C6H10O	98.1	845	341.958	1.52227
(*E*)-2-Hexenal monomer	C6728263	C6H10O	98.1	845.9	343.046	1.187
1,8-Cineole dimer	C470826	C10H18O	154.3	1039.4	668.172	1.72879
1,8-Cineole monomer	C470826	C10H18O	154.3	1039.9	669.06	1.29601
1-Menthol dimer	C2216515	C10H20O	156.3	1164.6	899.447	1.8906
1-Menthol monomer	C2216515	C10H20O	156.3	1167.1	904.068	1.21812
1-Octen-3-one	C4312996	C8H14O	126.2	980.7	559.797	1.2695
1-Propanol	C71238	C3H8O	60.1	567.8	133.475	1.11258
2-Butanone dimer	C78933	C4H8O	72.1	598.6	148.067	1.24992
2-Butanone monomer	C78933	C4H8O	72.1	600.2	148.809	1.06203
2-Methyl butanol dimer	C137326	C5H12O	88.1	730.6	226.066	1.47232
2-Methyl butanol monomer	C137326	C5H12O	88.1	732.1	227.383	1.22414
2-Methylbutanal dimer	C96173	C5H10O	86.1	661.8	177.992	1.4041
2-Methylbutanal monomer	C96173	C5H10O	86.1	659.7	177.003	1.16061
2-Pentenal (*E*) dimer	C1576870	C5H8O	84.1	745.3	238.45	1.36565
2-Pentenal (*E*) monomer	C1576870	C5H8O	84.1	745.6	238.713	1.10984
2-Pentylfuran	C3777693	C9H14O	138.2	1001.9	598.781	1.24128
3-Methylbutanal dimer	C590863	C5H10O	86.1	652.4	173.54	1.41084
3-Methylbutanal monomer	C590863	C5H10O	86.1	649.7	172.304	1.17493
3-Octanol dimer	C589980	C8H18O	130.2	983.5	565.022	1.77447
3-Octanol monomer	C589980	C8H18O	130.2	984.2	566.228	1.38555
5-Methyl-2-furanmethanol dimer	C3857258	C6H8O2	112.1	954.5	511.571	1.5706
5-Methyl-2-furanmethanol monomer	C3857258	C6H8O2	112.1	955.8	513.982	1.2601
6-Methyl-5-hepten-2-one	C110930	C8H14O	126.2	994.7	585.518	1.18325
Acetone	C67641	C3H6O	58.1	541.7	121.109	1.12438
Benzaldehyde	C100527	C7H6O	106.1	955	512.563	1.15226
Butyl acetate	C123864	C6H12O2	116.2	804.6	294.952	1.24116
Ethyl 2-methylbutanoate	C7452791	C7H14O2	130.2	841.7	338.13	1.66084
Ethyl 2-methylpropanoate	C97621	C6H12O2	116.2	749.8	242.27	1.56892
Ethyl butyrate	C105544	C6H12O2	116.2	800.4	290.033	1.20896
Heptanal dimer	C111717	C7H14O	114.2	899.4	410.268	1.70124
Heptanal monomer	C111717	C7H14O	114.2	899.3	409.926	1.32837
Heptanoic acid dimer	C111148	C7H14O2	130.2	1053	693.3	1.763
Heptanoic acid monomer	C111148	C7H14O2	130.2	1051.6	690.583	1.36001
Hexanal dimer	C66251	C6H12O	100.2	791.1	279.29	1.56594
Hexanal monomer	C66251	C6H12O	100.2	791.1	279.29	1.2557

Isoamyl acetate dimer	C123922	C7H14O2	130.2	876	378.144	1.74648
Isoamyl acetate monomer	C123922	C7H14O2	130.2	876.6	378.846	1.3001
Linalool oxide dimer	C60047178	C10H18O2	170.3	1076.6	736.767	1.81745
Linalool oxide monomer	C60047178	C10H18O2	170.3	1077	737.673	1.26043
Methyl 2-methylbutanoate dimer	C868575	C6H12O2	116.2	768.8	258.259	1.54017
Methyl 2-methylbutanoate monomer	C868575	C6H12O2	116.2	770	259.284	1.18366
Pentanal dimer	C110623	C5H10O	86.1	694.6	195.765	1.42769
Pentanal monomer	C110623	C5H10O	86.1	694.9	196.046	1.17999
Propylacetate dimer	C109604	C5H10O2	102.1	707.7	206.831	1.48212
Propylacetate monomer	C109604	C5H10O2	102.1	708.4	207.358	1.16971
Propylsulfide	C111477	C6H14S	118.2	884.7	388.206	1.16255

- represents volatile compounds that not annotated in the database.
